# Exogenous Oct-4 Inhibits Lens Transdifferentiation in the Newt *Notophthalmus viridescens*

**DOI:** 10.1371/journal.pone.0102510

**Published:** 2014-07-14

**Authors:** Rital B. Bhavsar, Panagiotis A. Tsonis

**Affiliations:** Department of Biology and Center for Tissue Regeneration and Engineering, University of Dayton, Dayton, Ohio, United States of America; National Cancer Institute, United States of America

## Abstract

From the cocktail of four factors that were able to induce pluripotent stem cells from differentiated cells, Oct-4, c-Myc, Sox-2 and Klf4, only Oct-4 was not expressed during regeneration in newts. To explore the possible action of this stemness factor we developed an assay where we introduced exogenous Oct-4 protein to an *in vitro* system for lens regeneration in newts. We found that exogenous Oct-4 inhibits differentiation of iris pigmented epithelial cells into lens cells and also regulates Sox-2 and Pax-6, both important players during lens development. Thus, presence of Oct-4 hinders transdifferentiation of iris cells.

## Introduction

The ability to regenerate lost body parts in certain organisms is either due to resident stem cells or dedifferentiation of tissues at the site of injury. In newt, the regenerated lens is derived from pigmented epithelial cells of dorsal iris by the process of transdifferentiation [1]. Thus, iris pigmented epithelial cells (IPE) change their lineage to form cells of the regenerated lens. Similar event of changing lineage occurs during *in vitro* reprogramming of terminally differentiated cells. In mammalian reprogramming, differentiated cells are forced to change their lineage or revert back to undifferentiated state by exogenous expression of tissue specific transcription factors or stem cell maintenance factors [2]. Studies have found similarities between these two processes as observed in regeneration animal models such as newt, zebrafish and frogs [3]–[5]. These similarities are mainly related to common gene expression.

In newts, regulated expression of stem cell factors; Sox-2, c-Myc and Klf4 is observed during both lens and limb regeneration [4]. In particular for lens regeneration expression was studied at the early phases spanning the first 10 days after lentectomy. At day 4 cells of the iris re-enter the cell cycle. By day 8 a depigmented dedifferentiated vesicle has formed at the dorsal iris. Sox-2 showed higher expression in the ventral iris at day 2 and 8, while Klf-4 did not show any notable regulation in both dorsal and ventral iris. c-Myc steadily increased from day 0 to day 8 in both irises most likely correlated with proliferation and dedifferentiation. The same set of genes is also expressed during zebrafish fin regeneration [3]. In addition, Oct-4 is expressed and required during zebrafish fin regeneration unlike regeneration in newts. Oct-4 has been a crucial pluripotency factor in reprogramming of various cell types to pluripotent cells [6]. Regenerating tissue exhibits restricted potential by changing its fate to only those cell types that are lost during injury. The absence of Oct-4 during regeneration of lens and limbs in newts poses the obvious question of what would be the role of exogenous Oct-4 during regeneration?

Understanding mechanisms that are involved in stem cell reprogramming and underlying principles of *in vivo* regeneration will provide insights into common events occurring during cell fate changes. Here, we describe our findings for the consequences of Oct-4 expression in newt IPE cells to understand cellular fate change during lens regeneration.

## Results and Discussion

To examine the effects of exogenous Oct-4 in newt regenerating tissue we used a recombinant protein delivery system combined with a newly established *in vitro* system of studying lens regeneration in newts [7]. According to this system, dorsal and ventral iris epithelial are dissociated and placed in culture. While in culture certain treatments can take place, such as with growth factors, with certain pathway-inducing or –activating molecules, or transfecting exogenous genes. After that, the cells are re-aggregated and either placed in a lentectomized eye or embedded in matrigel. The result of such culturing protocol is that, as it happens in the *in vivo* conditions, only the dorsal aggregate can be transdifferentiated to lens and not the ventral. Thus, this is an excellent and robust assay to find out the effects of factors or genes in inducing the ventral iris cells to transdifferentiate to lens or in inhibiting transdifferentiation of the dorsal iris cells. This protocol is much better from other protocols involving re-implantation of the aggregates in a letectomized eye. First it is much faster requiring 1–2 weeks to ascertain the results, while re-implantation take 4–5 weeks in which period loss of animals could be a major factor. Second, transfection of genes or proteins as in the present case is much more efficient allowing for better evaluation of the data.

**Figure 1:**
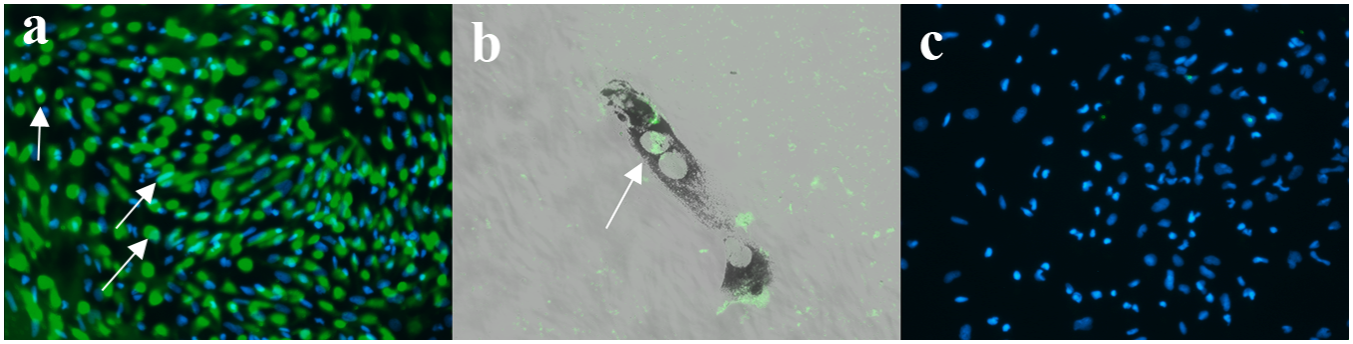
Newt IPE cells treated with Oct-4 protein. a) IPE cells after 7 hours of protein treatment (earliest time to visualize Oct-4 protein). Cells (arrow) show Oct-4 protein (green) transfer to nucleus (blue). b) Confocal image merged with bright field showing Oct-4 (green) localization in the cells at higher magnification. c) IPE cells not treated with oct-4 antibody show only nuclear stain (blue). The efficiency of oct-4 translocation increases at prolonged incubation time points (data not shown).

**Figure 2:**
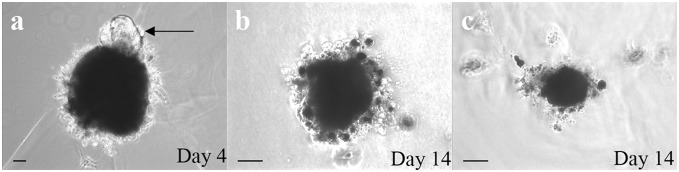
Live images of IPE cell aggregates placed on matrigel. a) Dorsal control aggregate showing transdifferentiated transparent lentoid (arrow) on day 4 of matrigel treatment. b) Dorsal Oct-4 protein treated aggregate without lentoid formation at day 14 on matrigel. c) Ventral Oct-4 protein treated aggregate without lentoid formation at day 14 on matrigel. Scale bar = 50 µm.

**Figure 3:**
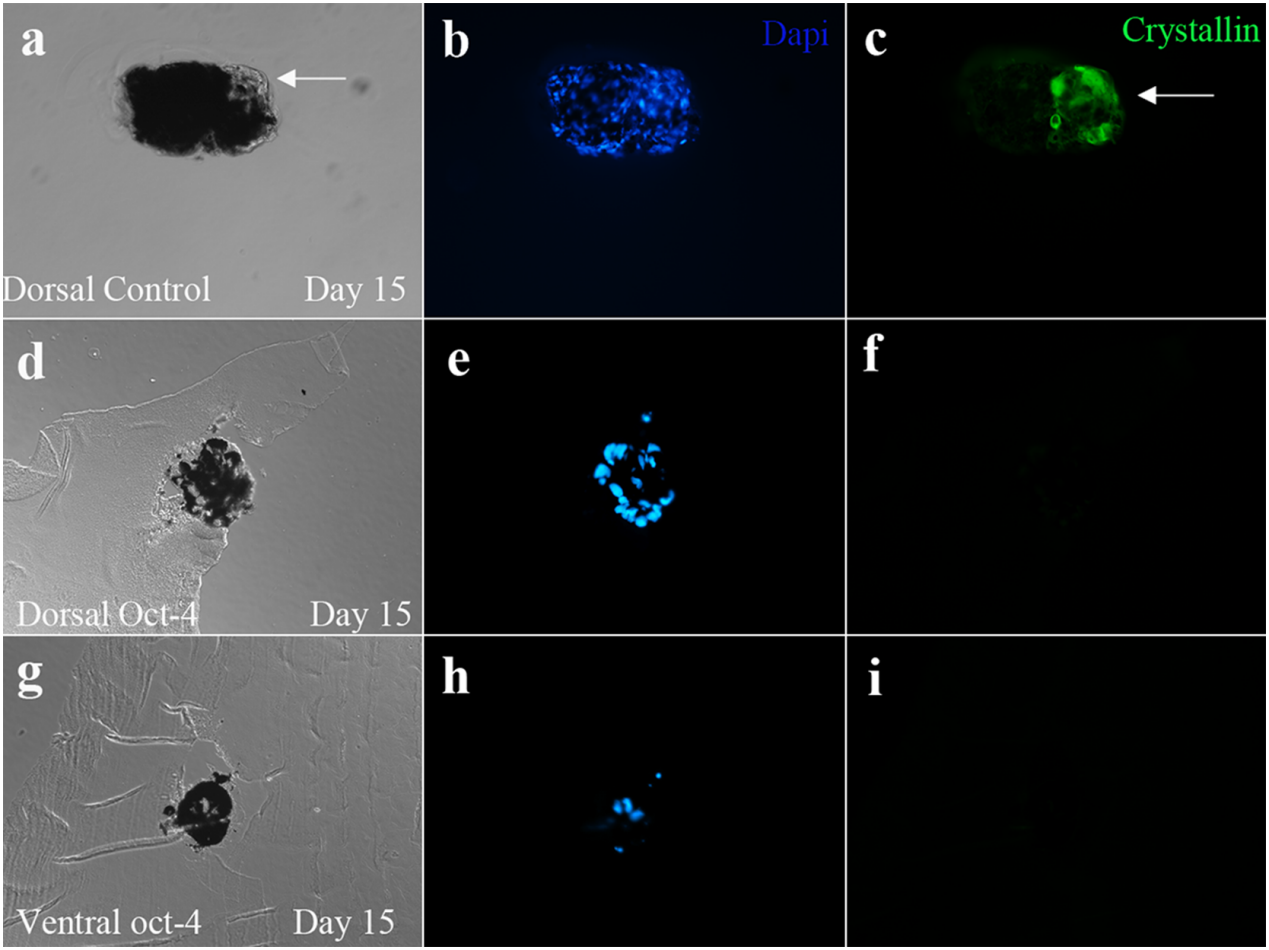
Immunohistochemistry of aggregates placed on matrigel. Control dorsal aggregate showing transdifferentiated transparent lentoid (arrow, a) and positive for crystallin expression (c). Dorsal Oct-4 treated aggregate negative for crystallin expression (d, f). Ventral Oct-4 treated aggregate negative for crystallin expression (g, i). Control dorsal aggregate image was captured using confocal microscope. The lentoid in the control aggregates was observed from day 4 to day 15 of matrigel treatment. Oct-4 treated aggregates were sectioned into 10 µm sections for staining. Nuclear staining of aggregates (b, e and h).

**Figure 4:**
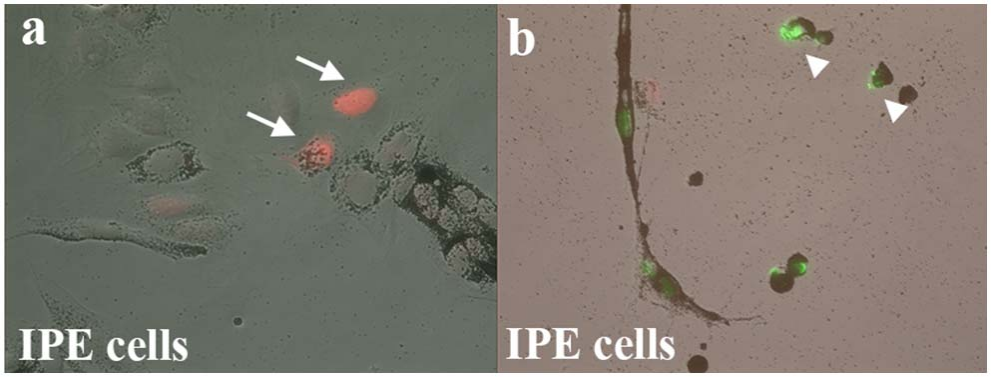
Dual staining of transfected IPE cells with Oct-4 (red) and Tunel (green). a) Cells expressing Oct-4 (arrows) are Tunel-negative. b) Cells positive for Tunel (arrowheads) are Oct-4-negative.

In order to express exogenous Oct-4 in cultured newt IPE cells, cells were treated with recombinant human Oct-4 protein as described in previous studies [8]. We decided to use the protein (instead of a plasmid) to increase the efficiency and provide us with more reliable results. Within 7 hours of treatment, Oct-4 protein translocated to the nucleus (figure 1a). This is the earliest time point to detect Oct-4 localization in cell as in agreement with previous study [8]. Traces of Oct-4 could be also seen in the cytoplasm (figure 1b). The Oct-4 treated cells when aggregated and placed on matrigel displayed reduced potential for transdifferentiation (Table 1). Thus, Oct-4 presence inhibited transdifferentiation of these aggregates. Only 10% of aggregates were able to form lentoids as compared to 81% by control aggregates. Figure 2 shows a control dorsal aggregate with lentoid formation at day 4 on matrigel (a) and Oct-4 treated aggregates lacking lentoid formation (b and c). The controls as well as Oct-4 treated aggregates were observed for 2 weeks on matrigel. To confirm lentoid formation, aggregates were stained for alphaA crystallin, a marker for lens. Aggregates exhibiting transdifferentiation expressed crystallin (figure 3a–c). Crystallin expression was not observed for aggregates that failed to transdifferentiate (figure 3d–i). These results are unlikely to be because of cells death. As shown in Figure 4 dual staining of transfected cells with Oct-4 and Tunel clearly shows that expression of Oct-4 is not associated with cell death.

**Table 1:** *In vitro* matrigel assay results for IPE cell aggregates treated with Oct-4 protein.

IPE cells Aggregate	Treatment	Aggregate showingtransdifferentiationon matrigel	Percentage oftransdifferentiatedaggregates
Dorsal	Oct-4 protein	1 in 10	10%
Ventral	Oct-4 protein	0 in 14	0%
Dorsal	Control	22 in 27	81%
Ventral	Control	0 in 22	0%

IPE- Iris Pigmented Epithelial.

The aggregates were placed on matrigel and observed for transdifferentiation (lentoid formation) for a period of 14 days.

**Figure 5:**
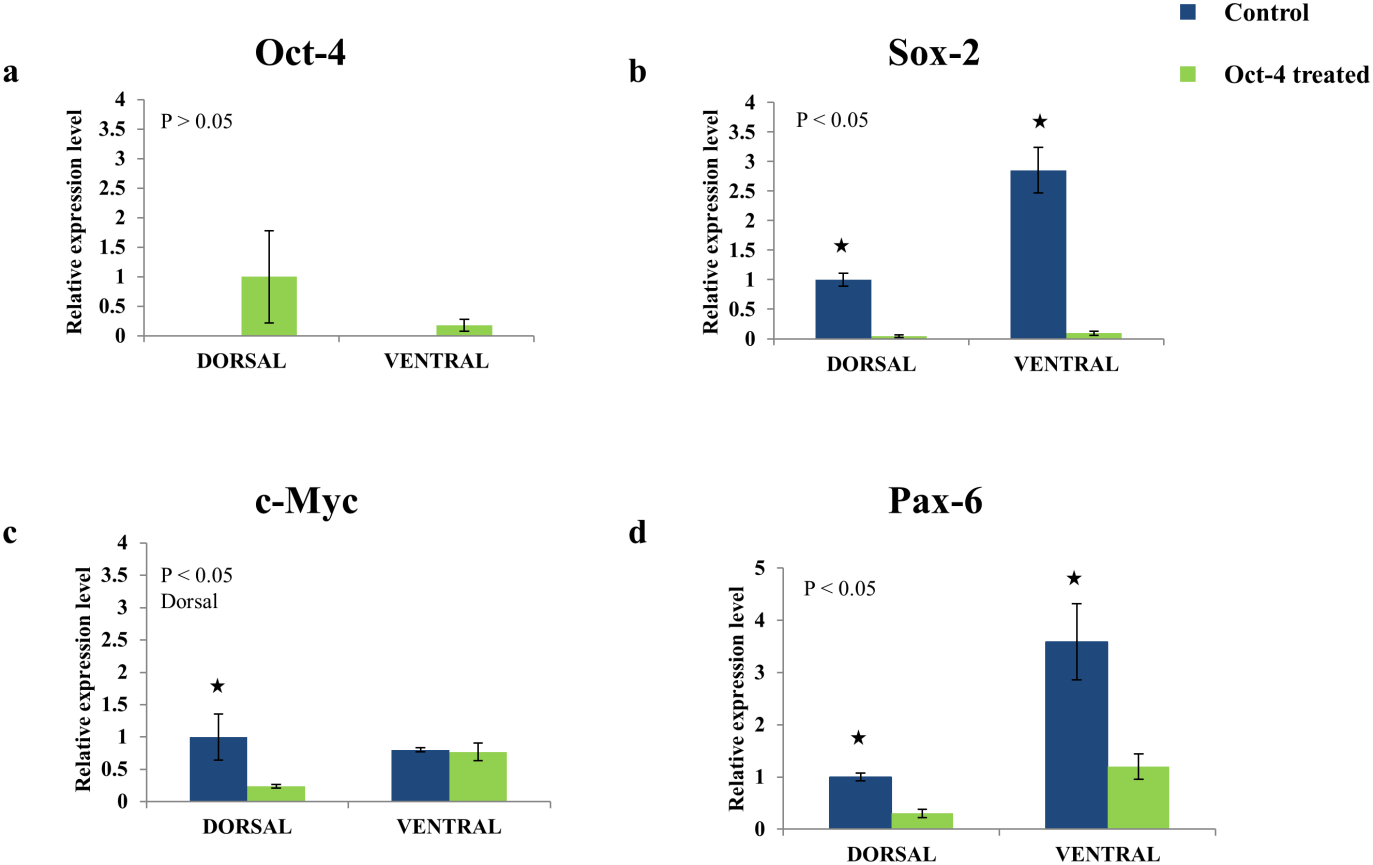
mRNA expression analysis of a) Oct-4 b) Sox-2 c) c-Myc d) Pax-6 in newt dorsal and ventral IPE cells with or without Oct-4 protein treatment. The control samples were cells not treated with Oct-4 protein. P value was obtained by anova analysis for Sox-2, Pax-6 and c-Myc. For oct-4 expression, p value was calculated by independent t-test for oct-4 treated groups. *Groups show significant difference between the control and oct-4 treated cells.

Further, we examined expression of other stem cell pluripotency factors after protein treatment. In dorsal control IPE cells, Oct-4 and Klf4 are not expressed whereas Sox-2 and c-Myc are expressed (figure 5). The absence of Oct-4 is totally consistent with the in vivo data (Maki et al, 2009). Klf-4 was not found despite several trials. The reason for this could be that even in vivo Klf-4 was the one factor with the lowest expression and not really correlated with the early events of regeneration (cell cycle re-entry and dedifferentiation). And since we are dealing with cultured cells this expression could have been diminished. Sox-2 showed more expression in the ventral cells and c-myc was virtually equal in ventral and dorsal cells, patterns which are similar to the in vivo results. In Oct-4-treated cells, Oct-4 expression was observed in both dorsal and ventral iris along with genes Sox-2 and c-Myc (figure 5a–c). Expression of Oct-4 means that the protein regulates its own gene as well. However, Sox-2 was significantly down-regulated as compared to the control cells (figure 5b). Sox-2 has been identified as an important factor in pluripotency maintenance. During reprogramming or ES cell maintenance, Oct-4 and Sox-2 co-occupy many developmentally regulated genes to suppress differentiation [9]. Contrary to this, our study showed that Oct-4 has a negative effect on Sox-2 expression. This could be possible because of cell type specific role of Sox-2 during lineage establishment. In eye, Sox-2 has been an interacting partner with pax-6 for crystallin synthesis [10]. Pax-6 is an eye specific transcription factor whose expression is required for lens regeneration and crystallin synthesis [11]. In relation to this we examined expression of pax-6 in our system. We found that similarly to Sox-2, pax-6 was significantly down regulated in Oct-4-treated newt IPE cells (figure 5d). This could explain our data because both pax-6 and Sox-2 are paramount for lens fiber differentiation during lens development and regeneration. Oct-4 is also shown to affect c-Myc expression in dorsal IPE cells (figure 5c). c-Myc being a growth regulator [12] and its down regulation in dorsal IPE cells further supports the inhibitory effect of Oct-4 on transdifferentiation.

In summary, we found that Oct-4 affects the transdifferentiation process of newt IPE cells during lens regeneration. The loss of transdifferentiation also correlated to changes in gene expression signatures for transcription factors Sox-2 and Pax-6 whose role is crucial for lens formation.

## Methods

### Ethics Statement

Animal care measures were strictly followed as described by Institutional Animal Care and Use Committee. The protocol was approved by the committee on research ethics and compliance of the University of Dayton (Protocol ID: 011-02). Animal surgeries were performed under 0.1% ethyl 3-aminobenzoate-methane sulfonic acid anesthesia, and all efforts were made to minimize suffering.

### Culture of newt IPE cells

Newts (*Notophthalmus viridescens*) were obtained from Sullivan and Co., Inc, Tennessee. For each culture, 7 newts were anesthetized in 0.1% ethyl 3-aminobenzoate-methane sulfonic acid prepared in PBS. Eyes were removed and placed in CMF (calcium and magnesium free) hanks solution. Dorsal and Ventral IPE cells were isolated from each eye and plated on collagen I plates [13]. The plates were incubated at 27**°**C. On day 8 onwards, cells were fed with fresh L15 medium every alternative day. It should be noted here that transdifferentiation is a default state of the cultured dorsal iris cells but it takes long time and only lentoids are formed. However, treatment of these cells with factors could enhance and speed-up their transdifferentiation process.

### Oct-4 protein treatment of newt IPE cells

Recombinant human Oct-4 11-R was purchased from LD Biopharma (HTF-0006). Total 4 cycles of Oct-4 protein treatment (8 µg/ml of L15 medium) were given to each well of cultured dorsal and ventral IPE cells: cycle 1-day 9, cycle 2-day 11, cycle 3-day 13 and cycle 4-day 15. Cells were treated with protein over night and changed to fresh medium on next morning. A lag period of 36 hours was kept between each cycle period [8].

### Immunocytochemistry and Immunohistochemistry

Cultured newt IPE cells were treated with Oct-4 protein for 7 hours. This was the earliest time point for detecting oct-4 localization in cells as described by the Zhou group [8]. Following treatment, the medium was removed and cells were washed with PBS before fixation with 4% PFA (paraformaldehyde). Cells were blocked with 10% goat serum and incubated with Oct-4 antibody (1∶100 dilution; stemgent) at 4**°**C overnight. Goat anti rabbit Alexflour 488 (1∶100 dilution) and Dapi were used to detect Oct-4 and nuclei respectively. Images were captured using fluorescence and confocal microscopy for low and high magnification respectively. Cell apoptosis was analyzed by millipore fluorescein TUNEL assay for adherent IPE cells transfected with Oct-4 as per manufacture’s instructions and was followed by staining for Oct-4. Anti-rabbit Cy3 (1∶100 dilution) secondary antibody was used for Oct-4 detection.

For immunohistochemistry of IPE aggregates, the aggregates were fixed in 4% PFA and embedded in paraffin. The embedded aggregates were sectioned at 10 µm thickness using microtome. The sections were then deparaffinized and hydrated before blocking with 10% goat serum. Mouse anti-alphaA antibody was used to detect crystallin presence in the sectioned aggregates. Goat anti mouse Alexflour 488 was used as secondary antibody. Nuclei were stained using Dapi. Images were captured using fluorescence microscope.

### QPCR

RNA was isolated from both dorsal and ventral Oct-4 protein treated cells (at day 18 in culture) using Trizol (ambion) and concentrated using RNA concentrator kit from zymo research as per directions in the respective manuals. For control samples, untreated cells were used for RNA isolation. cDNA was synthesized using first strand synthesis kit from GE health care as per directions. QPCR was performed with iQSYBR green supermix (biorad). The primers used were: Oct-4-F: TGCAATCGTCGACAGAAGGG; Oct-4-R: AGCATGGTTGGCAAGGCATA; Sox-2-F: GCGCAGGGATACATGAACGG; Sox-2-R: AGTGCGAAGATGACGAGGTG; c-Myc-F: CAACCGGAAGTGCACAAGTC; c-Myc-R: GCCACCTCTGGTATCTGGTC; klf4-F: CGGACGGCTACCCATAACTG; klf4-R: AGTGATAGGGCTTCTCGCCT; pax6-F: GAATGTACGACAAGCTGCGG; pax6-R: GGAGTTGGTGTTCTCGCCTC; RPL27-F: ATTTATGAAACCCGGGAAGG; RPL27-R: CCAGGGCATGACTGTAAGGT. Three technical replicates were used for each gene and Ct values were calculated using standard curve. Specificity of QPCR reaction was checked by melt curve analysis. Statistical analysis was performed using independent t-test (oct-4) or univariate ANOVA (Sox-2, c-Myc and Pax-6). Gene expression levels were normalized using RPL27 [14].

### Sequencing of PCR products

To further verify Oct-4 activation in the treated cells, the PCR products for its reaction were purified from the gel. A sequencing reaction was performed using big dye terminator v3.1 sequencing kit (applied biosystems). PCR steps used were: 1) 96**°**C for 10 seconds 2) 95**°**C for 10 seconds; 55**°**C for 5 seconds; 60**°**C for 4 minutes (30x). The DNA was precipitated using ethanol and samples were analyzed using ABI Capillary Electrophoresis Genetic Analyzer 3130. Sox-2 products were also verified by sequencing.

### *In vitro* Matrigel Assay

To prepare cell aggregates, on day 17 cells were treated with dispase. Next day, cells were dislodged by pipetting and collected in a micro centrifuge tube. The cells were washed with L15 medium twice. In the final step, 200 ul of L15 media was added to the cell pellet and spinned for 2 minutes at 1000 rpm. The tubes were incubated at 27**°**C for 48 hours for cell aggregation. From each well, 2 aggregates were prepared. For *in vitro* matrigel assay, 100 µl of matrigel was placed in a form of drop on tissue culture treated plates [7]. The plates were incubated at 37**°**C for 30 minutes to solidify the matrigel. The aggregate was held in a pipette tip along with some L15 medium and placed inside the upper edge of matrigel. Each matrigel-aggregate arrangement was provided with 2 ml of L15 medium. The plates were incubated at 27**°**C and medium was changed every 2 days. The aggregates were checked for transdifferentiation (lentoid formation) from day 2 until 2 weeks by observing under light microscope. Images were taken using light microscopy.
